# Supplementary material for: Quantifying trade-offs between therapeutic efficacy and resistance dissemination for enrofloxacin dose regimens in cattle
Source: Sci Rep. 2024 Sep 4;14:20598. doi: 10.1038/s41598-024-70741-8 (PMC11374901; doi:10.1038/s41598-024-70741-8)

Title: **Quantifying trade-offs between therapeutic efficacy and resistance dissemination for enrofloxacin dose regimens in cattle**

Authors: Liton Chandra Deb[1*], Archana Timsina[1], Suzanne Lenhart[2], Derek Foster[1], and Cristina Lanzas[1]

Supplementary Figure 1: Uncertainty of the states: (**a**) susceptible of *E. coli* (S_e), (**b**) susceptible of *P. multocida* (S_m), (**c**) resistant of *E. coli* (R_e), and (**d**) resistant of *P. multocida* (R_m). These distributions for the ODE solutions of model (1) when the parameter set is varied according to the uniform distributions given in Table 2. In this case, the treatment is 7.5 mg/kg single dose. The dark red curves represent the average solution curves of the states obtained from the uncertainty distribution. Other light pink, pink, and dark pink ribbons represent the solutions in 25-75 quantiles of the uncertainty distribution of states.

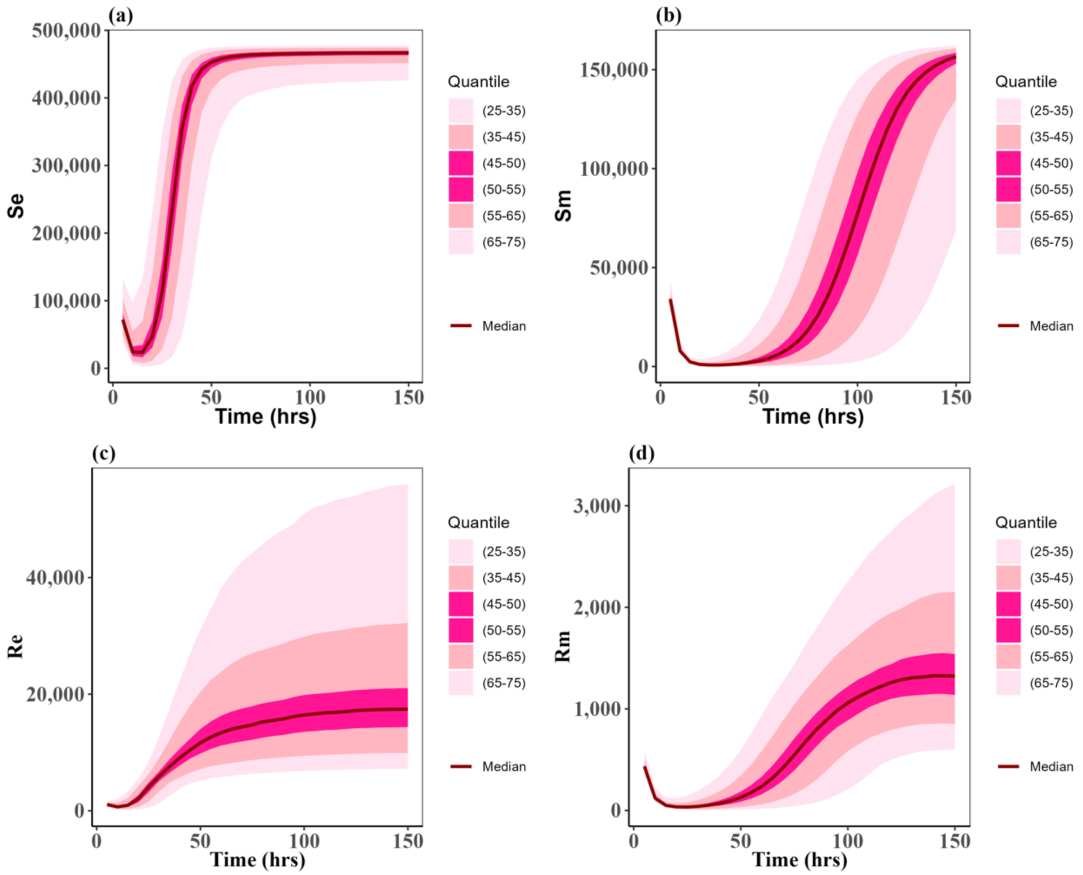

Supplement: Supplementary file 1 — Supplementary Figure 1. [file 41598_2024_70741_MOESM1_ESM.pdf]
